# Effectiveness of a Participatory and Culturally Tailored Learning Program for Coronavirus Prevention in Muslim Older Adults With Hypertension and Diabetes: Quasi-Experimental Study

**DOI:** 10.2196/71671

**Published:** 2025-10-01

**Authors:** Sutteeporn Moolsart, Choochat Phuangsomjit

**Affiliations:** 1 School of Nursing, Sukhothai Thammathirat Open University, 9/9 Mhu 9, Bang Phut Subdistrict, Pak Kret District, Nonthaburi, 11120, Thailand, 660860318420; 2 School of Education, Sukhothai Thammathirat Open University, 9/9 Mhu 9, Bang Phut Subdistrict, Pak Kret District, Nonthaburi, Thailand

**Keywords:** Thailand, participatory and culturally based, learning program, coronavirus, older adults, Muslim

## Abstract

**Background:**

Respiratory infections have increased globally over time, with older adults being the most susceptible demographic. Programs based on cultures and experiences strongly correspond with useful, real-world applications.

**Objective:**

This study evaluated a participatory and culturally based learning program’s effect on awareness, knowledge, and preventive behaviors regarding coronavirus and health care among Muslim older adults with hypertension and diabetes in Thailand.

**Methods:**

The quasi-experimental study used a 2-group pretest-posttest design with participants aged 60‐80 years with hypertension and diabetes. The sample included 35 in the experimental group and 32 in the control group. An interactive 6-week learning program focusing on coronavirus prevention and health care was developed by incorporating Muslim cultural perspectives and Kolb experiential learning model, alongside standard care. The control group received only standard care. Data were collected via a questionnaire covering general information, awareness, knowledge, and preventive behaviors related to coronavirus prevention and health care. The content validity indices of sections II, III, and IV were 0.98, 0.99, and 0.96, respectively. The Cronbach α coefficients of awareness and behaviors were 0.89 and 0.86, respectively, while the KR-20 for knowledge was 0.86. Data analysis was conducted using the *t* test, Mann-Whitney *U* test, and Wilcoxon signed-rank test.

**Results:**

The results indicated that, after participating in the program, the awareness, knowledge, and preventive behaviors related to coronavirus and health care among older adults in the experimental group significantly improved compared to their preprogram levels and to those in the control group, with a *P* value <.01.

**Conclusions:**

The program effectively improved coronavirus prevention and health care among Muslim older adults. It could be broadly applied in similar contexts and to other severe respiratory diseases.

## Introduction

During the coronavirus pandemic, older adults were particularly vulnerable and faced multiple challenges, including limited access to health care, increased isolation, and emotional distress [1,2]. Muslim older adults were considered a high-risk group for coronavirus infection due to their regular participation in communal religious activities, such as weekly mosque prayers, physical greetings, and daily worship rituals [3]. In Muslim-majority countries such as India, Sri Lanka, and Bangladesh, significant numbers of coronavirus infection cases were reported [4]. In Thailand, although the Sheikhul Islam issued preventive guidelines [5], tensions arose between religious practices and public health recommendations [6]. These included dilemmas regarding mosque attendance, social distancing, and following imams via online platforms. Understanding the cultural and spiritual beliefs of Muslims is essential for delivering effective and respectful health care. Older Muslims, particularly those with limited access to digital media, often receive inadequate health information, which can result in poor compliance with preventive measures.

Older adults often live with chronic conditions such as hypertension, diabetes, and cardiovascular disease, which significantly increase their risk of severe outcomes from coronavirus infection [7,8]. A systematic review reported that individuals with these conditions have a 2- to 4-fold higher risk of severe illness and mortality [9]. Despite this evidence, many studies have overlooked the specific needs and perspectives of high-risk older adults, particularly those with multimorbidity [10].

Promoting coronavirus prevention education among older adults with comorbidities was essential [11]. Thailand’s cultural and religious diversity, particularly in provinces such as Nonthaburi, added complexity to public health efforts. Nonthaburi reported a high number of coronavirus infection cases during the outbreak, with Muslims constituting the second-largest religious group after Buddhists. A pilot study conducted in Pak Kret District, Nonthaburi Province, Thailand, surveyed 124 Muslim households and revealed that 64.5% (80/124) of older adults had hypertension and 39.5% (49/124) had diabetes. Despite demonstrating high levels of awareness and knowledge, strong preventive behaviors, and moderate motivation to learn, 45.2% (56/124) of participants reported having contracted coronavirus. Notably, knowledge (*r*=0.543) and willingness to learn (*r*=0.465) were significantly correlated with preventive behavior. Awareness, however, showed a small nonsignificant correlation (*r*=0.126) at *P>*.01. Between 2020 and 2022, many Muslim older adults were surrounded by individuals with limited knowledge and inadequate preventive practices [12]. Therefore, interventions should target awareness, knowledge, and behavior at the individual, family, and community levels to promote sustainable and appropriate health practices in older adults with comorbidities.

Enhancing participatory and culturally grounded learning was essential for promoting coronavirus prevention among vulnerable Muslim older adults [13,14]. A learner-centered approach, tailored to their cultural context, helps increase receptiveness to new knowledge. Understanding their beliefs and lived experiences fosters trust and facilitates the exchange of skills, supporting long-term improvements in awareness, knowledge, and preventive behavior—even in the event of a future outbreak.

However, a literature review revealed a lack of prevention programs specifically designed for this group. Most existing studies focus on Thai Buddhist older adults and emphasize knowledge enhancement [15] and coronavirus health literacy [16-18]. These programs typically last 4 to 12 weeks and involve lectures, skill training, media use, group activities, self-management, and LINE-based knowledge sharing (LINE Corporation). Results consistently show significant improvements in preventive behavior.

Internationally, technology-based services such as mobile health and telehealth have been shown to benefit older adults by supporting treatment, information access, self-monitoring, and counseling [19,20]. Yet, prior research has predominantly focused on individual-level interventions. More attention should be paid to environmental and social influences, as infection is often spread to older adults by people in their immediate surroundings.

This study aimed to evaluate the effectiveness of a participatory and culturally sensitive learning program for coronavirus prevention and health care among vulnerable Muslim older adults in Thailand with comorbid hypertension and diabetes. The program was designed based on David A. Kolb's experiential learning cycle—experience, reflection, concept, and application [21]—and was integrated with Muslim cultural values. Understanding how Islamic beliefs influence health practices is essential in providing effective care for Muslim patients. Nurses and health care providers must be culturally competent and sensitive to these beliefs in order to deliver equitable care. This approach not only fosters individual potential but also promotes social equity for vulnerable older adults. In the long term, culturally grounded participatory learning programs may help prevent severe respiratory viral infections among ethnic older populations with chronic diseases.

## Methods

### Study Design

The study design was quasi-experimental, specifically a 2-group pretest-posttest design. The research was carried out for 6 weeks to investigate the effectiveness of the participatory and culturally based learning program for coronavirus prevention plus usual standard care for older adults with comorbid hypertension and diabetes. The study was registered at the Thai Clinical Trials Register (TCTR20250112007).

### Study Participants

The sample group consisted of Muslim older individuals with comorbid hypertension and diabetes. It was divided into 2 groups: the experimental group, which lived in Tha It Subdistrict, Pak Kret District, and the control group, residing in Lahan Subdistrict, Bang Bua Thong District, Nonthaburi Province. The 2 areas were selected through simple random sampling from 19 Muslim communities of Nonthaburi province. Both subdistricts had comparable contextual characteristics.

To determine the sample size, the G*power program [22] was used to estimate the effect size from a study on the impact of a health literacy promotion program on disease prevention behaviors among older Thai Buddhists in Bangkok [17] and Phra Nakhon Si Ayutthaya [18], which yielded effect sizes of 4.52 and 3.02. This resulted in sample sizes of 7 and 14 for each group. Therefore, for this study, the effect size was estimated at 0.90, using a *t* test for the difference between 2 independent means (2 groups), with an alpha error probability of .05 and a power (1 – β error probability) of .95. The program calculated a sample size of 56, resulting in 28 individuals per group. To prevent sample loss, the researcher increased the sample size by 40%, resulting in 40 individuals per group.

Systematic random sampling was used to recruit older adults from the community. Eligible individuals were invited to participate in the program by community health volunteers, who visited every second household (ie, skipping one house) in the community.

Inclusion criteria were Muslim older adults aged 60‐80 years with comorbid hypertension and diabetes, residing in Tha It Subdistrict, Pak Kret District, or Lahan Subdistrict, Bang Bua Thong District, Nonthaburi Province, both male and female, without cognitive impairments, capable of understanding and communicating in Thai, and without significant thinking problems or depression. Exclusion criteria included participation for less than 6 consecutive weeks, prior coronavirus infection, serious illness preventing participation, or withdrawal from the program.

### Research Tools

The experimental implementation tool consisted of a participatory and culturally based learning program to prevent coronavirus disease and enhance health care for older adults. This program was a set of activities aimed at controlling the factors that caused the spread of coronavirus disease and self-care for health, with a learner-centered approach. It focused on using the learning-by-doing method as the foundation for developing the learning process. It drew out the learners’ abilities and encouraged them to participate in teaching activities and think critically to solve problems. Second, a team of researchers thoughtfully developed the coronavirus prevention and health care manual. The content of food and exercise was adapted to Muslims. Third, videos titled “7 Steps of Hand Washing,” “Chair Exercises,” “Food for Hypertension and Diabetes,” and “Stress Control,” publicly shared on YouTube (Google LLC), were applied. Fourth, material and equipment such as masks, food models, salt meters, and PowerPoint (Microsoft Corp).

The data collection tool included a questionnaire on awareness, knowledge, and preventive behaviors regarding the coronavirus disease for older adults, which was developed by the researchers. It consisted of 4 sections: (1) general information: this section consisted of open-ended and closed-ended questions, totaling 15 items. (2) Awareness of preventing coronavirus infection and health care consisted of 15 items. The questions were a 5-point rating scale of least aware, somewhat aware, moderately aware, very aware, and most aware. (3) Knowledge of preventing coronavirus infection and health care, assessing memory and understanding of preventing coronavirus infection and health care, with 20 multiple-choice questions. The questions were in the format of correct-uncertain-incorrect. The interpretation of knowledge was divided into 3 levels according to the concept of Bloom et al [23] as follows: below 60% (0.00‐11.00 points) was little, 60%‐79.99% (12.00‐15.00 points) was moderate, and 80% and above (16.00‐20.00 points) was very good. (4) Preventive behaviors against coronavirus and health care, divided into 5 areas: prevention of coronavirus, dietary habits, exercise, stress management, and medication adherence, totaling 40 items. The questions were in the form of a 5-point rating scale: never practiced at all, practice occasionally, occasionally practice, almost always practice, and practice regularly. The interpretation of awareness and behaviors was least aware or practice (1.00‐1.50), somewhat aware or practice (1.51‐2.50), moderately aware or practice (2.51‐3.50), very aware or practice (3.51‐4.50), and most aware or practice (4.51‐5.00).

The participatory learning program and the questionnaire were validated by 5 experts in nursing, public health, medicine, or evaluation. The content validity indices of the awareness, knowledge, and behavior sections were 0.98, 0.99, and 0.96, respectively. The Cronbach α coefficients of awareness and behaviors were 0.89 and 0.86, respectively, while the KR-20 for knowledge was 0.86.

### Interventions

**Figure 1.**
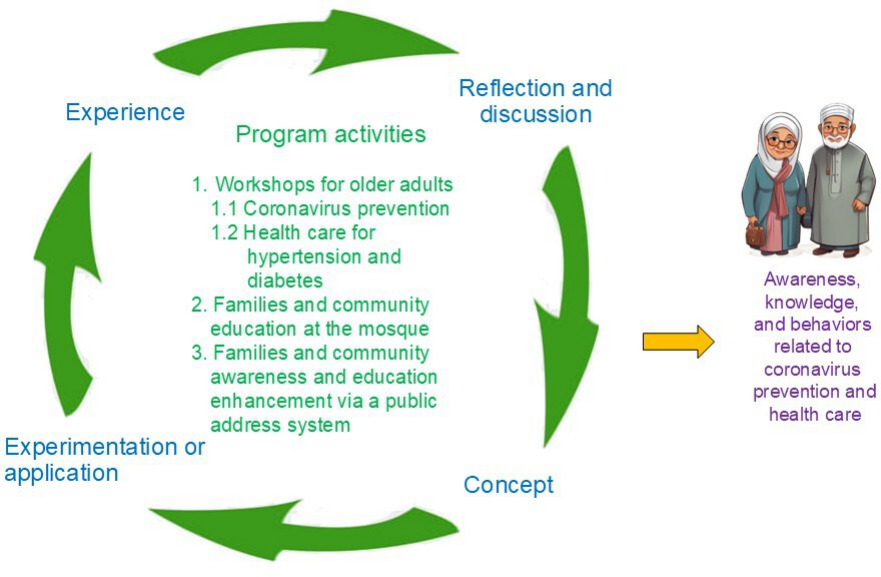
A participatory and culturally based learning program for coronavirus prevention and health care for Muslim older adults with hypertension and diabetes.

The program activities were based on Muslim culture and Kolb steps: experience, reflection and discussion, concept, and experimentation or application (Figure 1). The activities included 3 main components managed by the researcher: first, workshops were conducted for the experimental group of older adults at the Darul Aman School meeting room in Tha It Subdistrict, Pak Kret District, Nonthaburi Province. The 6-week program included workshops conducted 3 times, specifically on Fridays during the first, fourth, and sixth weeks, prior to the participants attending Friday prayers. The details of the workshop activities are presented in Table 1. In this table, each week began with a review and compilation of participants’ information and prior experiences.

**Table 1.** Workshop activities in a participatory and culturally based learning program for coronavirus prevention and health care among Muslim older adults.

Program activities		Duration (min)
Week 1		
	Lecture: risk of coronavirus infection in older adults	30
	Experience sharing and reflection on coronavirus prevention	30
	Health care for coronavirus prevention	30
	Mask selection and wearing training	30
Week 4		
	Experience sharing and reflection on the application of coronavirus prevention in daily life	30
	Antigen Test Kit (ATK) testing training	30
	Creating nutritious diet plans	45
	Exercise training	15
Week 6		
	Strengthening application knowledge about coronavirus prevention	30
	Understanding coronavirus vaccines	30
	Encouraging self-care commitment	30

Second, community education was conducted over a 6-week period, once a week on Fridays after prayers, from 1:30 PM to 2 PM (30 min per session), totaling 6 sessions. The aim was to prevent coronavirus infections within families and communities, particularly by protecting older adults and ensuring proper care for individuals with hypertension and diabetes. At the mosque, both the Imam and professional nurses—respected figures within the Muslim community—delivered sessions to raise awareness and disseminate health knowledge among individuals, families, and the broader community.

Third, community and family awareness were further enhanced using a public address system over the same 6-week period. Broadcasts were made twice a week, every Monday and Wednesday morning (10‐15 min per session), totaling 12 sessions. These broadcasts aimed to provide concise education on coronavirus prevention and health care management for individuals with chronic diseases and their families, promoting the integration of preventive behaviors into daily life.

Usual care for coronavirus prevention, provided by government employees, included the DMHTT strategy: social distancing (D), mask-wearing (M), handwashing (H), temperature checking (T), and testing (T). Medication, nutrition, exercise, mood management, and abstinence from alcohol and tobacco were the mainstays of care for diabetes and hypertension. Both the control group and the experimental group were subjected to these strategies.

### Data Collection

After proceeding to request an ethics certificate for research involving human participants from the School of Nursing, Sukhothai Thammathirat Open University, the researcher requested permission from the Islamic Religious Organization, the Public Health Office, the District Municipality, and the Subdistrict Administrative Organization to conduct research in the setting. Coordinate with the subdistrict health promotion hospital to conduct the research in a sample group of older adults with hypertension and diabetes as an experimental and control group according to the inclusion criteria, totaling 40 people each. Work with Darul Aman School to set up the space for the 6-week program for the older adults that would be held there from January 13 to February 24, 2023. Nurses and local health volunteers served as study assistants and collected the data.

### Statistics Analysis

The data were analyzed using SPSS software (IBM Corp). General information, awareness, knowledge, and preventive behaviors were analyzed with descriptive statistics, including frequency, percentage, mean, and SD. The pre-experimental data were tested for normal distribution using the Kolmogorov-Smirnov test, following the assumptions of *t* test statistics. The variables of awareness and preventive behaviors were normally distributed, while knowledge did not follow a normal distribution. As a result, a nonparametric test was used to compare differences in knowledge data.

### Ethical Considerations

This study was approved by the Ethical Committee of the School of Nursing, Sukhothai Thammathirat Open University (Reference No. NS 1/2566, dated January 12, 2023) and registered with the Thai Clinical Trials Registry (TCTR20250112007). All participants provided written informed consent after being informed about the study’s objectives, procedures, potential risks and benefits, confidentiality measures, and their rights as research participants. Participation was voluntary, and individuals were given sufficient time to consider their involvement and discuss it with their families. Participants were provided with transportation allowance and refreshments, including snacks and lunch, during study activities. They were assured of their rights to withdraw at any time without consequence. All collected data were kept strictly confidential and used solely for research purposes in accordance with institutional and international ethical guidelines.

## Results

### Overview

**Figure 2.**
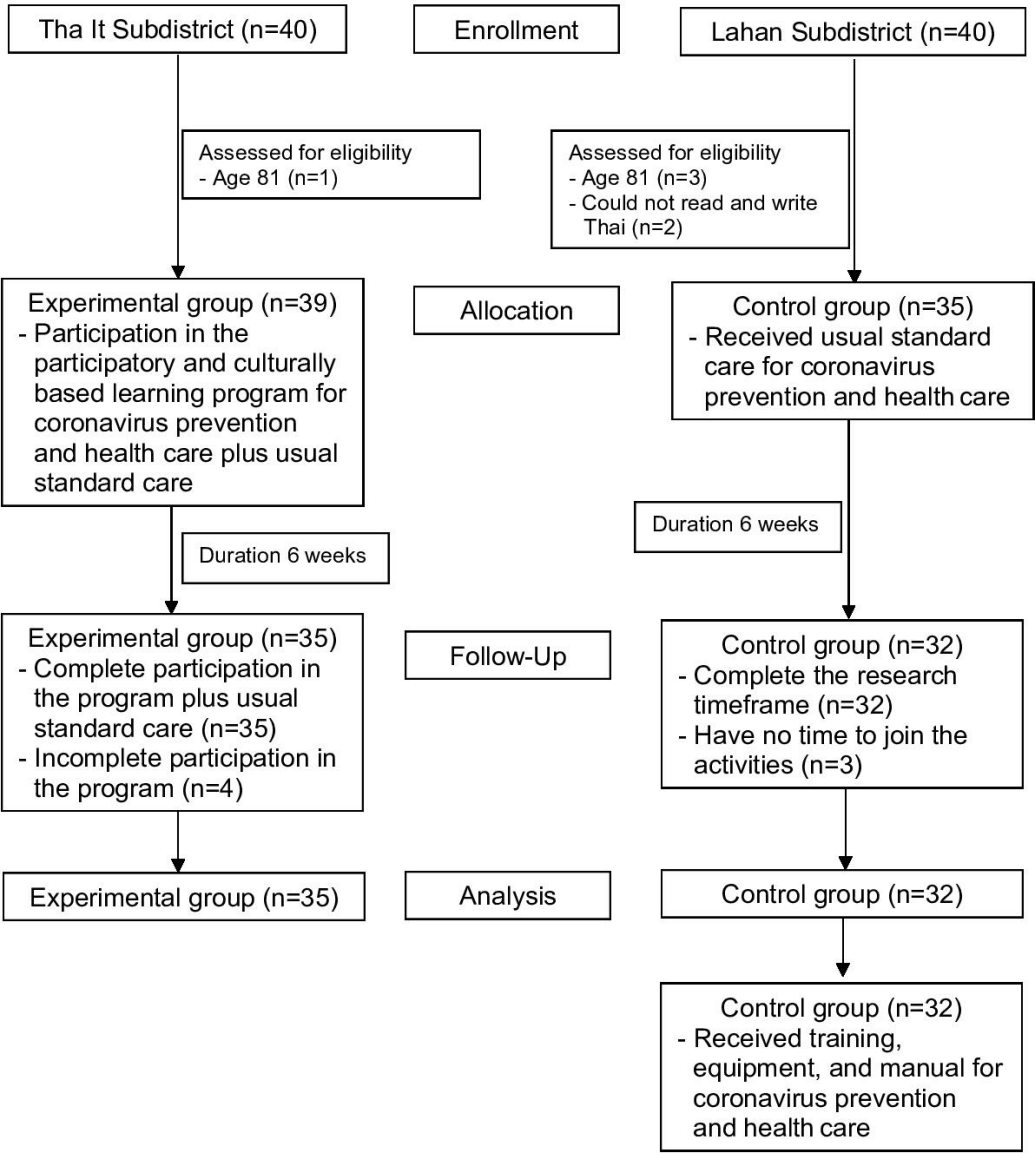
A Consolidated Standards of Reporting Trials flow diagram of selecting the participants in the study.

A total of 74 older adults who met the inclusion criteria attended the program as scheduled, with 39 participants assigned to the experimental group and 35 participants to the control group. At the end of the 6-week program, retention rates were 89.7% (n=35) in the experimental group and 91.4% (n=32) in the control group (Figure 2).

### Sociodemographic and Background of Participants

The demographic and health characteristics of participants are presented in Table 2. The majority of the experimental group was female (77%, 27/35) compared to 66% (21/32) in the control group. The average age was similar between groups (66.11 years in the experimental group and 68.25 years in the control group), with most participants aged 60‐65 years. Most were married and had completed primary education. A small proportion in the experimental group (9%, 3/35) had no formal education. Most participants were unemployed, though the experimental group had more employed individuals, mainly as casual laborers or housemaids.

**Table 2.** Demographic and health characteristics of the experimental and control groups.

Demographic and health characteristics	Experimental group		Control group		Chi-square or *t* test (*df*)	*P* value
Sex, n (%)					*χ*²=1.092 (1)	.30
Male	8 (23)		11 (34)			
Female	27 (77)		21 (66)			
Age (years), mean (SD; range)	66.11 (4.70; 60-76)		68.25 (4.74; 60-79)		*t*=1.850 (65)	.07
Age group (years), n (%)						
60‐65	19 (54)		10 (31)			
66‐70	7 (20)		11 (35)			
71‐75	8 (23)		10 (31)			
75‐80	1 (3)		1 (3)			
Marital status, n (%)					*χ*²=0.322 (1)	.57
Single or widow or separate	14 (40)		15 (47)			
Couple	21 (60)		17 (53)			
Education, n (%)					*χ*²=4.037 (2)	.13
Uneducated	3 (9)		0 (0)			
Primary level	29 (82)		26 (81)			
Over than primary level	3 (9)		6 (19)			
Occupation, n (%)					*χ*²=12.319 (1)	≤.01^a^
Unemployed	15 (43)		27 (84)			
Employed	20 (57)		5 (16)			
Duration of hypertension, mean (SD; range)	8.18 (4.34; 1-20)		12.17 (8.99; 2-45)		*t*=2.266 (65)	.03
Duration of hypertension, n (%)						
Less than 5 years	11 (31)		9 (28)			
6‐10 years	18 (52)		10 (31)			
More than 10 years	6 (17)		13 (41)			
Duration of diabetes, mean (SD; range)	8.03 (5.87; 1-20)		12.10 (9.07; 1-45)		*t*=2.187 (65)	.03
Duration of diabetes, n (%)						
Less than 5 years	15 (43)		9 (28)			
6‐10 years	12 (34)		10 (31)			
More than 10 years	8 (23)		13 (41)			
Family experience with coronavirus infection, n (%)					*χ*²=2.364 (1)	.12
Never	15 (43)		8 (25)			
Having experiences	20 (57)		24 (75)			
Participant’s experience with coronavirus infection, n (%)					*χ*²=0.558 (1)	.46
Never	19 (54)		14 (44)			
Having experiences	16 (46)		18 (56)			

a *P*<.01.

The majority in both groups had been living with hypertension and diabetes for 6‐10 years, with the control group having had the conditions for about 4 years longer on average. Over half of the families in both groups had experienced coronavirus infection (57% in the experimental group and 75% in the control group), and nearly half of the older adults themselves had been infected. There was a statistical difference in occupation as well as the duration of hypertension and diabetes between the experimental and control groups at P<.01.

### The Comparison of Awareness, Knowledge, and Behavior Related to Coronavirus Prevention and Health Care

Before participating in the program, the experimental group had significantly lower mean knowledge scores than the control group (*P*<.01). However, after the intervention, the experimental group showed significantly higher knowledge scores compared to the control group (*P*<.01; Table 3).

**Table 3.** Comparison of mean knowledge, awareness, and preventive behaviors regarding coronavirus prevention and health care between the experimental and control groups at pre- and postprogram participation.

Variables	Experimental group (n=35)		Control group (n=32)		*Z/t* ^a^	*P* value
	Mean (SD)	Level	Mean (SD)	Level		
Knowledge						
Preprogram participation	12.68 (0.36)	Moderate	16.75 (0.92)	Good	−6.135	<.01^b^
Postprogram participation	16.34 (1.73)	Good	14.97 (3.03)	Moderate	−3.157	<.01^b^
Awareness						
Preprogram participation	3.94 (0.36)	Good	4.03 (0.49)	Good	−0.834	.41
Postprogram participation	4.42 (0.44)	Good	4.08 (0.54)	Good	2.896	<.01^b^
Behaviors						
Preprogram participation	3.57 (0.38)	Good	3.96 (0.19)	Good	−5.360	<.01^b^
Postprogram participation	4.17 (0.45)	Good	3.87 (0.46)	Good	2.647	.01^b^

a The Mann-Whitney *U* test was used to compare knowledge scores, while independent *t* tests were used to compare awareness and behavior scores.

b *P*<.01.

Regarding awareness, there was no significant difference between the experimental and control groups before the program. However, the experimental group had significantly lower behavior scores than the control group at baseline (*P*<.01). Following the intervention, the experimental group demonstrated significantly higher levels of both awareness and preventive behaviors compared to the control group (*P*<.01; Table 3).

After participating in the program, the experimental group demonstrated a significant increase in knowledge regarding coronavirus prevention and health care compared to their preprogram scores. In contrast, the control group showed a significant decrease in mean knowledge scores (*P*<.01; Table 4).

**Table 4.** Comparison of mean knowledge, awareness, and preventive behaviors related to coronavirus prevention and health care before and after program participation in the experimental and control groups.

Variables	Preprogram participation		Postprogram participation		*Z/t* ^a^	*P* value
	Mean (SD)	Level	Mean (SD)	Level		
Knowledge						
Experimental group	12.68 (0.36)	Moderate	16.34 (1.73)	Good	−4.276	<.01^b^
Control group	16.75 (0.92)	Good	14.97 (3.03)	Moderate	−4.139	<.01^b^
Awareness						
Experimental group	3.94 (0.36)	Good	4.42 (0.44)	Good	5.033	<.01^b^
Control group	4.03 (0.49)	Good	4.08 (0.54)	Good	0.456	.65
Behaviors						
Experimental group	3.57 (0.38)	Good	4.17 (0.45)	Good	5.680	<.01^b^
Control group	3.96 (0.19)	Good	3.87 (0.46)	Good	−0.836	.41

a The Wilcoxon signed-rank test was used to compare knowledge scores, while paired *t* tests were used to compare awareness and behavior scores*.*

b *P*<.01.

In addition, the experimental group exhibited significant improvements in both awareness and preventive behaviors related to coronavirus following the intervention (*P*<.01). Meanwhile, the control group showed no significant changes from their preprogram levels (Table 4).

## Discussion

### Principal Findings

This study demonstrated that a participatory and culturally based learning program significantly enhanced awareness, knowledge, and preventive behaviors related to coronavirus and chronic disease care among vulnerable Muslim older adults in Thailand. The program was developed using Kolb experiential learning theory, which emphasized active participation through concrete experience, reflective observation, abstract conceptualization, and active experimentation [21]. The improved outcomes in the experimental group highlight the value of culturally tailored and learner-centered approaches in health promotion.

The knowledge, awareness, and practice included proper mask use, handwashing, ATK testing, DASH and low-carb diet selection, and tailored exercises such as arm swings and chair-based movements to strengthen calf muscles and improve glucose metabolism. While this study did not assess blood glucose levels or overall health outcomes, previous research demonstrated that activating the soleus muscle through low-intensity contractions while sitting significantly reduced postprandial glucose and insulin levels [24], suggesting that such localized muscular activities may support metabolic health in older adults with diabetes.

In alignment with prior studies [15-18,25], the inclusion of group discussion, skill-building, and culturally congruent practices fostered deeper engagement and behavior change. Moreover, the involvement of trusted community leaders—particularly the Imam—strengthened message acceptance, consistent with findings from diabetes education programs in Muslim communities [25,26].

A unique strength of this study was its integration of religious, cultural, and familial structures into the learning process. By conducting workshops in mosques before or after prayer and using public broadcasting systems in the community, the intervention reached not only the participants but also their families and neighbors. This expanded reach likely supported knowledge dissemination and encouraged family members to reinforce preventive practices at home, in line with previous studies showing the importance of social support in influencing older adults’ behaviors [27]. Also, the literature found that the level of understanding and positive information evaluation status associated with coronavirus protective behaviors of the Chinese older adults [28].

Although coronavirus is no longer a global emergency, the threat of future outbreaks of respiratory viruses remains. This program serves as a prototype for adapting culturally based, community-led interventions to promote preventive behaviors for other infectious diseases, such as influenza, respiratory syncytial virus, or emerging zoonoses. The program’s experiential and reflective nature also lends itself well to modifying content while maintaining the underlying structure, especially for communities where cultural values shape health behaviors.

However, careful adaptation is essential. For instance, video content must be reviewed for cultural appropriateness, background music should be avoided, and prayer times respected. Postprayer educational sessions must be concise due to time constraints. Furthermore, Islamic rituals—such as bowing during worship—cannot be altered, though modifications such as using a cloth or alternative greetings were accepted by some older adults. These findings underscore the need for an interdisciplinary, culturally sensitive approach. As Attum et al [26] emphasized, Muslims view health as a gift from God, with spiritual well-being often prioritized alongside physical health. This perspective shapes their response to illness and health interventions. Songwathana et al [29] similarly found that Muslim older adults grounded their health behaviors in spiritual meaning and a belief in living naturally and peacefully in old age.

### Study Limitations

This study was conducted in a specific Muslim community in Thailand, and thus, cultural and environmental factors may limit its generalizability to other regions or religious groups. Participants were selected by geographical clusters, which may have introduced selection bias. In addition, the program did not assess clinical outcomes such as blood pressure, blood glucose levels, or BMI, limiting insights into long-term health impacts.

### Implications for Nursing Practice

Nurse practitioners and primary care teams can implement participatory and culturally responsive health education programs tailored to older adults with chronic diseases. Such programs should be extended over a longer period to allow for the measurement of clinical outcomes and behavior sustainability. Moreover, they can be adapted to address other infectious diseases in multicultural settings, particularly those affecting vulnerable populations with limited health access. This approach aligns with the expanded role of nurses in health promotion, community engagement, and chronic disease management during and beyond the coronavirus pandemic [30,31].

### Conclusions

Participatory and culturally based learning programs have positively impacted older adults with hypertension and diabetes by enhancing their awareness, knowledge, and behaviors regarding coronavirus prevention and health care. Implementing similar initiatives in communities across Thailand could contribute to the long-term prevention of severe respiratory diseases.

## Supplementary material

10.2196/71671 Checklist 1 CONSORT checklist.

## References

[1] Lebrasseur A, Fortin-Bédard N, Lettre J (2021). Impact of the COVID-19 pandemic on older adults: rapid review. JMIR Aging.

[2] Cosco TD, Fortuna K, Wister A, Riadi I, Wagner K, Sixsmith A (2021). COVID-19, social isolation, and mental health among older adults: a digital catch-22. J Med Internet Res.

[3] (2021). Minimizing COVID-19 risk around religious celebrations as European region sees resurgence of cases. World Health Organization.

[4] (2021). SEAR weekly situation report #24. World Health Organization.

[5] (2020). Sheikhul Islam office announces COVID-19 measures for mosques. ASEAN NOW.

[6] Narongraksakhet I, Salaming M, Kaewtubtim M, Al-Usamah A, Jehte R (2022). Culture and beliefs of Thai Muslims in southern border provinces of Thailand in protecting and reducing the spreading of the virus corona disease 2019 by Islamic teachings, health principles and local wisdoms. J Islamic Stud.

[7] Duru S (2020). COVID-19 in elderly patients. Eurasian J Pulmonol.

[8] Tadic M, Cuspidi C (2021). The influence of diabetes and hypertension on outcome in COVID‐19 patients: do we mix apples and oranges?. J Clin Hypertens.

[9] de Almeida-Pititto B, Dualib PM, Zajdenverg L (2020). Severity and mortality of COVID 19 in patients with diabetes, hypertension and cardiovascular disease: a meta-analysis. Diabetol Metab Syndr.

[10] Richardson SJ, Carroll CB, Close J (2020). Research with older people in a world with COVID-19: identification of current and future priorities, challenges and opportunities. Age Ageing.

[11] (2023). Risk of severe COVID-19 increases with the number of comorbidities in fully vaccinated individuals aged ≥65: results from INFORM. British Geriatrics Society.

[12] Kasatpibal N (2023). Overcoming the COVID-19 crisis: a social analysis for tackling future pandemics. Khonthai40.

[13] Dassieu L, Sourial N (2021). Tailoring interventions for social isolation among older persons during the COVID-19 pandemic: challenges and pathways to healthcare equity. Int J Equity Health.

[14] Tierney S, Wong G, Mahtani KR (2021). Unpacking culture and the well-being of older people during the COVID-19 pandemic. Oxford Social Prescribing Research Network.

[15] Moongtavekait W, Vichean P, Chawalitsuphaserani P, Hundee O, Wongsaree C (2021). Effective of knowledge program on self-care preventive behavior of COVID-19 among elderly in Prathumthani Province. PTUJ.

[16] Matthapa K (2021). Effect of health literacy enhancement program on preventive behaviors of coronavirus 2019 among elderly in That Municipality Warincharab district, Ubon Ratchathani Province. RHP 10 Ubon.

[17] Sornsnam W, Ruamsook T, Tipwong A, Passara R, Kaewtong N (2022). Effects of health promoting model on health literacy and COVID-19 preventive behaviors among the elderly in elderly club in Bangkok Metropolis. Thai J Nurs.

[18] Werunat M, Moolsart S, Tipkanjanaraykha K (2022). The effectiveness of a health literacy development program in coronavirus 2019 prevention for older adults with diabetes mellitus in Phra Nakhon Si Ayutthaya Province. J R Thai Army Nurses.

[19] Abbaspur-Behbahani S, Monaghesh E, Hajizadeh A, Fehresti S (2022). Application of mobile health to support the elderly during the COVID-19 outbreak: a systematic review. Health Policy Technol.

[20] Haimi M, Gesser-Edelsburg A (2022). Application and implementation of telehealth services designed for the elderly population during the COVID-19 pandemic: a systematic review. Health Informatics J.

[21] Kolb DA (1984). Experiential Learning: Experience as the Source of Learning and Development.

[22] Faul F, Erdfelder E, Buchner A, Lang AG (2009). Statistical power analyses using G*Power 3.1: tests for correlation and regression analyses. Behav Res Methods.

[23] Bloom BS, Madaus GF, Hastings JT (1971). Handbook on Formative and Summative Evaluation of Student Learning.

[24] Hamilton MT, Hamilton DG, Zderic TW (2022). A potent physiological method to magnify and sustain soleus oxidative metabolism improves glucose and lipid regulation. iScience.

[25] Odglun Y, Sranacharoenpong K, Nirdnoy N (2023). Effect of the culturally tailored diabetes prevention program for at-risk Thai Muslim people in semi-urban areas. J Health Res.

[26] Attum B, Hafiz S, Malik A, Shamoon Z (2023). StatPearls.

[27] Manart C, Kerdmonkol P, Amnartsatsue K (2023). Factors related to preventive behaviors towards coronavirus 2019 among older adults in Nonthaburi Province. Public Health Nurs.

[28] Sun Z, Yang B, Zhang R, Cheng X (2020). Influencing factors of understanding COVID-19 risks and coping behaviors among the elderly population. Int J Environ Res Public Health.

[29] Songwathana P, Chinnawong T, Ngamwongwiwat B (2023). Health practice among Muslim homebound older adults living in the southern border provinces of Thailand. Bulitung Nurs J.

[30] Moolsart S (2020). Roles of nurse practitioners in the prevention and control of the epidemic of coronavirus disease 2019 in the community. Public Health Nurs.

[31] Moolsart S (2020). The trend of care management for multicultural patients with chronic diseases in Thailand post-pandemic world of coronavirus disease 2019. Thai J Nurs.

